# Statin Use and the Presence of Microalbuminuria. Results from the ERICABEL Trial: A Non-Interventional Epidemiological Cohort Study

**DOI:** 10.1371/journal.pone.0031639

**Published:** 2012-02-16

**Authors:** Arjan van der Tol, Wim Van Biesen, Steven Van Laecke, Kris Bogaerts, Koen De Lombaert, Hans Warrinnier, Raymond Vanholder

**Affiliations:** 1 Renal Division, Department of Internal Medicine, University Hospital Ghent, Ghent, Belgium; 2 Interuniversity Centre for Biostatistics and Statistical Bioinformatics, Leuven, Belgium; 3 F. Hoffman-La Roche Ltd, Brussels, Belgium; University of British Columbia, Canada

## Abstract

**Background:**

Microalbuminuria (MAU) is considered as a predictor or marker of cardiovascular and renal events. Statins are widely prescribed to reduce cardiovascular risk and to slow down progression of kidney disease. But statins may also generate tubular MAU. The current observational study evaluated the impact of statin use on the interpretation of MAU as a predictor or marker of cardiovascular or renal disease.

**Methodology/Principal Findings:**

We used cross-sectional data of ERICABEL, a cohort with 1,076 hypertensive patients. MAU was defined as albuminuria ≥20 mg/l. A propensity score was created to correct for “bias by indication” to receive a statin. As expected, subjects using statins vs. no statins had more cardiovascular risk factors, pointing to bias by indication. Statin users were more likely to have MAU (OR: 2.01, 95%CI: 1.34–3.01). The association between statin use and MAU remained significant after adjusting for the propensity to receive a statin based on cardiovascular risk factors (OR: 1.82, 95%CI: 1.14–2.91). Next to statin use, only diabetes (OR: 1.92, 95%CI: 1.00–3.66) and smoking (OR: 1.49, 95%CI: 0.99–2.26) were associated with MAU.

**Conclusions:**

Use of statins is independently associated with MAU, even after adjusting for bias by indication to receive a statin. In the hypothesis that this MAU is of tubular origin, statin use can result in incorrect labeling of subjects as having a predictor or marker of cardiovascular or renal risk. In addition, statin use affected the association of established cardiovascular risk factors with MAU, blurring the interpretation of multivariable analyses.

## Introduction

Microalbuminuria (MAU) is considered as a predictor or marker of cardiovascular morbidity and mortality, particularly in patients with other risk factors [1]–[4], and as a surrogate for early kidney damage especially in subjects with diabetes and hypertension [5], [6]. Statins are frequently prescribed in patients with hypertension, diabetes and metabolic syndrome, to reduce cardiovascular morbidity and mortality [7]. Statins can reduce existent proteinuria [8]–[10] through a positive impact on endothelial dysfunction. In contrast, there is in vitro [11], [12] and in vivo [13]–[16] evidence that statins are associated with de novo albuminuria and proteinuria. It is of importance to establish the association between statin use and MAU to correctly interpret presence of MAU as a predictor or marker of cardiovascular or renal disease in observational trials with a mixed population of subjects taking and not taking a statin. If statin use is associated with MAU, there is a risk of incorrect labeling of subjects as having a predictor or marker of cardiovascular or renal risk. The current study evaluated the association between statin use and MAU, and used a propensity score analysis to adjust for bias by indication. For this goal, we used the baseline data of the early renal impairment and cardiovascular assessment in Belgium (ERICABEL) trial, a prospective cohort of hypertensive patients followed by primary care physicians created to evaluate the impact of metabolic syndrome on cardiovascular and renal endpoints.

## Methods

### Objectives

The primary aim of the ERICABEL study was to determine the effect of metabolic risk factors on the evolution of renal function and cardiovascular outcome over 5 years, in patients aged between 40 and 70 years with diagnosed hypertension, and followed by their primary care physician. The current analysis was designed to evaluate 1° the association between statin treatment and MAU and 2° the impact of statin treatment on the interpretation of the association between individual cardiovascular risk factors and MAU in epidemiological studies.

### Participants

We used the baseline data of the ERICABEL cohort, a non-interventional epidemiological study with a follow up of 5 years that included 1,076 Caucasian patients with hypertension, defined as systolic blood pressure ≥140 mmHg and/or intake of at least one antihypertensive drug, recruited by 96 general practitioners, between 2006 and 2007, in Belgium. Of the 1076 patients included in this cross-sectional study, 420 patients had a missing value for at least one of the variables under investigation (see appendix table S1 for detailed list). Multiple imputation techniques were used to account for the missing data, using 20 imputations [17]. All characteristics and outcome (MAU) were simultaneously used in the imputation model. The imputation was done using the R function aregImpute from the Hmisc package [18].

### Description of procedures

Each participating primary care physician was asked to include 10 consecutive hypertensive patients aged between 40–70 years, in a 1∶1 sex ratio. The eligible persons were evaluated at baseline and if eligible, sociodemographic information (age, sex, race, and education level), personal and family medical history, smoking status and medication use were collected prospectively in an online database. Body weight was recorded to the nearest 0.1 Kg and height was measured to the nearest centimeter. Body mass index (BMI) was calculated as body weight in Kg divided by height^2^ (kg/m^2^). Blood pressure was measured according to the WHO criteria with a calibrated Omron HEM-907 device (average of 2 measurements, sitting, with 5 minutes in between). All these measurements were done by the primary care physician. After exclusion of a urinary infection or hematuria (negative Combur® test), MAU was screened by a Micral® dipstick test. MAU was considered present if measured albuminuria was ≥20 mg/l on a morning midstream urine sample. Blood sampling was performed by the general practitioner in fasting patients.

### Definitions

The metabolic syndrome was defined as three or more of the following criteria, according to the National Cholesterol Education Program Third Adult Treatment Panel guidelines ATP III criteria [19]: elevated blood pressure >130/85 mmHg and/or antihypertensive medication (by definition 100% in this cohort), (2) high plasma triglycerides (>1.7 mmol/l), low HDL cholesterol (<1.0 mmol/l in men and <1.3 mmol/l in women), (4) abdominal adiposity (waist circumference >102/88 cm men/women) and/or impaired glucose tolerance (IGT) (fasting plasma glucose >6.1 mmol/l and/or known diabetes).

### Ethics

The study was approved by an independent ethics committee review board, protocol number: ML 19208. A written informed consent was obtained from all participants.

### Statistical methods

All analyses have been performed using SAS software (SAS software, version 9.2 of the SAS System for Windows. Copyright © 2002 SAS Institute Inc.) or R Version 2.12.0 (R Foundation for Statistical Computing, Vienna, Austria. ISBN 3-900051-07-0, 2009) [20]. MAU was considered as a dichotomic variable. Continuous variables were described by their mean, standard deviation, median and interquartile range. Categorical variables were summarised by frequencies and percentages.

A propensity score for statin use was created to correct for “bias by indication”. Propensity score analysis is a well established method to adjust for confounding by indication in observational trials [21], [22]. Primarily, for the statistical analyses, 20 imputed samples were created. In a second step, within each of the 20 samples separately, a propensity model was constructed and the resulting propensity score was calculated for each of these patients. The propensity model included the following variables that were deemed to be possibly related to statin use: age, gender, BMI, waist circumference, SBP, previous CV event, CRP, fasting glucose, diabetes, serum uric acid, HDL and LDL cholesterol, triglycerides, use of angiotensin converting enzyme inhibitor/angiotensin receptor blocker (ACE-I/ARB) and smoking. Continuous variables in the model were included using restricted cubic splines: each continuous variable was included in the model using 3 dummy variables, called *var*1, *var*2 and *var*3. For the first 5 imputed samples, a histogram of the propensity scores was presented by statin use (see appendix table S2). In addition, in order to check the ability of the propensity scores to balance the two statin groups for baseline characteristics, tables were presented for the first 5 imputed samples, comparing the baseline characteristics between the groups (see appendix table S3). In this way, patients with the same “likelihood” or “propensity” to receive a statin (i.e. in this setting mainly with comparable cardiovascular risk factors), but in one case taking and in the other case not taking a statin, were compared for presence of MAU.

For comparison of continuous variables, ANOVA was used, adjusted for propensity scores, whereas for binary variables, logistic regression analyses, also adjusted for propensity scores, were employed. Logistic regression analyses were used to assess the association between statin use and MAU using the “GENMOD” procedure in SAS. The associations were assessed in each of the 20 imputation samples separately and the results were combined using the SAS procedure “MIANALYZE”. The following logistic regression models were used: 1°: Univariable model only including statin use; 2° a model including statin use and propensity scores (using a restricted cubic spline); 3° a model including statin use, propensity scores and all relevant variables mentioned above. Since the linearity assumption was deemed appropriate for all continuous variables in the model (p>0.1 for the assessment of linearity in the full model), the final model only included linear terms for all variables.

## Results

The baseline characteristics of the population are provided in tables 1 and 2. There was an equal distribution in gender (51.3% males) in the overall cohort. There was a high prevalence of metabolic syndrome (44.5%), diabetes (19.8%), current smokers (36.5%) and MAU (16.4%) in the overall cohort. ACE-I and/or ARB were the most commonly prescribed antihypertensive agents (55.9%). History of a cardiovascular event was recorded in 11.3% of the patients. Mean age of the cohort was 57.5±7.5 years. One third (30.8%) of the patients used a statin.

**Table 1:** Baseline characteristics (categorical variables).

parameter	No statins (N = 724)	Statins (N = 332)	Statin use unknown (N = 20)	Total (N = 1076)	P*	P§
**Female**	384/724 (53%)	132/332 (39.8%)	7/19 (36.8%)	523/1075 (48.7%)	<.001	<.001
**MS**	239/624 (38.3%)	172/299 (57.5%)	2/4 (50.0%)	413/927 (44.6%)	<.001	<.001
**Diabetes**	98/700 (14.0%)	104/327 (31.8%)	2/5 (40.0%)	204/828 (19.8%)	<.001	<.001
**Smoker**	249/698 (35.7%)	123/325 (37.9%)	3/5 (60.0%)	375/653 (36.5%)	0.438	0.501
**MAU**	68/529 (12.9%)	60/248 (24.2%)	0/4 (0.0%)	128/653 (16.4%)	<.001	<.001
**ACE-ARB**	358/724 (49.5%)	232/332 (69.9%)		590/1056 (55.9%)	<.001	<.001
**CV event**	38/693 (5.5%)	77/325 (23.7%)	1/5 (20.0%)	116/1023 (11.3%)	<.001	<.001

MS: metabolic syndrome, MAU: microalbuminuria, ACE-I/ARB: angiotensin converting enzyme inhibitor/angiotensin receptor blocker, CVevent: cardiovascular event.

p*:p values between all three groups; p§: p value between users vs. non statin users.

**Table 2:** Baseline characteristics (continuous variables).

Patient Characteristic	Statistic	No statin	Statin	Statin use unknown	Total	P*	P§
**Age (years)**	Mean±SD	56.8±7.6	59.1±7.1	58.2±6.0	57.5±7.5	<.001	<.001
	Median	57.2	60.0	58.6	58.5		
	N	724	332	19	1075		
**BMI (kg/m^2^)**	Mean±SD	29.3±5.4	30.6±5.5	30.5±4.4	29.7±5.4	0.001	<.001
	Median	28.5	29.7	31.7	28.8		
	N	689	326	5	1020		
**SBP (mmHg)**	Mean±SD	143.5±15.4	142.6±16.3	148.5±10.6	143.3±15.7	0.527	0.394
	Median	142.5	140.0	152.0	142.0		
	N	704	331	5	1040		
**DBP (mmHg)**	Mean±SD	84.4±9.7	82.9±9.9	86.7±5.8	84.0±9.8	0.054	0.020
	Median	84.5	82.0	86.5	83.5		
	N	704	331	5	1040		
**Glucose (mmol/l)**	Mean±SD	5.6±2.9	6.3±2.1	6.8±1.4	5.9±2.7	0.001	<.001
	Median	5.2	5.7	6.9	5.3		
	N	644	311	4	959		
**Uric acid (µmol/l)**	Mean±SD	339±89	357±83	291±83	345±89	0.008	0.004
	Median	333	357	286	339		
	N	629	306	4	939		
**Triglycerides (mmol/l)**	Mean±SD	1.6±1.1	1.9±1.1	2.0±1.7	1.7±1.1	0.011	0.003
	Median	1.3	1.6	1.4	1.4		
	N	638	316	4	958		
**LDL-Cholesterol (mmol/l)**	Mean±SD	3.2±0.8	2.7±1.0	2.9±0.8	3.0±0.9	<.001	<.001
	Median	3.2	2.6	3.2	3.0		
	N	628	312	4	944		
**HDL-Cholesterol (mmol/l)**	Mean±SD	1.5±0.5	1.4±0.4	1.3±0.4	1.5±0.5	0.004	0.001
	Median	1.4	1.3	1.4	1.4		
	N	637	315	4	956		
**CRP (mg/dl)**	Mean±SD	0.5±0.8	0.4±0.5	0.4±0.4	0.5±0.7	0.148	0.055
	Median	0.3	0.2	0.4	0.3		
	N	608	291	4	903		

BMI: body mass index, SBP: systolic blood pressure, DBP diastolic blood pressure, CRP: C-reactive protein. p*:p values between all three groups; p§: p value between users vs. non statin users.

In univariable analysis, statin users were more likely to be male (p<0.001), had a higher frequency of metabolic syndrome (p<0.001), diabetes type 2 (p<0.001), MAU (p<0.001), ACE-i/ARB treatment (p<0.001), and previous cardiovascular events (p<0.001), were older (p<0.001), had larger BMI (p<0.001), lower diastolic blood pressure (p = 0.02), higher fasting glucose (p<0.001), serum uric acid (p = 0.004), and triglycerides (p = 0.003), had lower levels of LDL (p<0.001) and HDL cholesterol (p = 0.001) compared to patients not taking statins (tables 1 and 2). The univariable odds ratio of MAU in patients using vs. not using a statin was 2.01 (95% CI: 1.34–3.01, p = 0.0009, table 3A).

**Table 3:** Association between statin use and microalbuminuria (MAU).

		Odds Ratio		
Logistic regression model for presence of MAU	Parameter	Estimate	95% Confidence Interval	P-value
A. Univariable association	Statin use	2.01	(1.34;3.01)	<0.001
B. Association adjusted for propensity score	Statin use	1.82	(1.14; 2.91)	0.01
	Pscore$	.		0.6
C. Association fully adjusted	Statin use	1.90	(1.15; 3.11)	0.01
	Pscore	7.92	(0.18; 357.3)	0.28
	Age	1.02	(0.91; 1.14)	0.74
	Diabetes	1.92	(1.004; 3.66)	0.05
	Smoker	1.49	(0.99; 2.26)	0.06
	ACE-I/ARB	1.47	(0.92; 2.34)	0.11
	CV event	1.32	(0.59; 2.95)	0.50
	BMI	1.03	(0.86; 1.24)	0.74
	Mean BP	0.96	(0.89; 1.04)	0.35
	Fasting glucose	0.97	(0.91; 1.04)	0.44
	Uric acid	0.89	(0.50; 1.50)	0.61
	Triglycerides	0.99	(0.98; 1.01)	0.44
	Cholesterol	1.00	(0.98; 1.02)	0.77
	CRP	1.49	(0.01; 359.73)	0.89

$ Pscore = propensity score; The propensity score was fit using a restricted cubic spline.

ACE-I/ARB: angiotensin converting enzyme inhibitor/angiotensin receptor blocker, CV event: cardiovascular event, BMI: body mass index, BP: Blood pressure, CRP: C-reactive protein.

After multivariable analysis including the propensity score for statin use, the odds of MAU was still significantly higher in patients taking a statin (OR: 1.82, 95% CI: 1.14–2.91, p = 0.01, table 3B). When all other variables were forced into the model, use of statin still was independently associated with a higher odds of MAU (OR: 1.90, 95% CI: 1.15–3.11, p = 0.01, table 3C). Next to statin use, only diabetes (OR: 1.92, 95% CI: 1.00–3.66, p = 0.05) and smoking (OR: 1.49, 95% CI: 0.99–2.26, p = 0.06) were independently associated with MAU after adjusting for the likelihood of receiving a statin, suggesting that prescription of a statin overrides the association between cardiovascular risk factors, MAU and creates collinearity by acting as a surrogate.

## Discussion

Our data create concern on the use of MAU as a predictor or marker of cardiovascular or renal disease in cohorts with patients using statins. Statin use apparently could blur the interpretation of MAU by two potential mechanisms: 1° higher prevalence of MAU in patients using a statin, even after correction for bias by indication and 2° masking of cardiovascular risk factors in multivariable analyses, as statin use behaves as a surrogate for these markers. In epidemiological studies evaluating the association between cardiovascular risk factors and MAU, statin use can induce incorrect labeling of patients as having a cardiovascular or renal risk factor, and interpretation of other risk factors for cardiovascular or renal disease can be confounded by the way the use of statins is handled in the analysis. In this cross-sectional analysis, we observed a two-fold higher prevalence of MAU in subjects who use vs. those who do not use a statin. However, part of this association (figure S1) can be attributed to bias by indication, as patients are often prescribed statins because they have cardiovascular risk factors which are by themselves associated with enhanced risk for MAU. Indeed, we observed a higher prevalence of cardiovascular risk factors in patients taking vs. not taking a statin in our study. We tried to exclude this bias by indication by the use of a propensity score analysis. Adjusting for the propensity score allows to analyze the difference in occurrence of MAU between patients with a comparable propensity to receive a statin, while one group does whereas the other does not receive the drug. The technique of propensity score is well established to address confounding and bias by indication in observational studies [23], [24]. However, this increased odds ratio remained present even after correcting for the fact that statins are usually prescribed in patients with cardiovascular risk factors which by themselves are associated with MAU, using the robust technique of propensity score. This observation can either be due to residual or unmeasured confounding or there can really be an induction of MAU by statin use (figure S1). Our data stress that statin use confounds the impact of the individual risk factors on MAU, as statin use behaves as a surrogate for presence of cardiovascular risk factors. As a consequence, in studies where MAU is either used as a marker or as a surrogate endpoint, the association between outcomes and certain cardiovascular risk factors can be blurred, and this in an unpredictable and variable fashion, depending upon the prevalence of statin use in the cohort. On the other hand, if statins really induce MAU, theoretically it can be both of glomerular or of tubular origin (figure S1). We did not find any publication, either human, animal or in vitro, indicating that statin associated proteinuria is of glomerular origin, but at least three in vitro or animal studies demonstrated that statins do inhibit tubular reabsorption of filtered albumin and in this way could generate MAU in a dose-dependent manner and in absence of cytotoxicity [11], [12], [25]. There is also growing evidence that also in other conditions MAU can be the consequence of tubular dysfunction, even in presence of an entirely intact glomerulus [26], [27]. One epidemiological study in humans also coined statin induced proteinuria as being tubular in origin, and even demonstrated a dose-effect relation with rosuvastatin [28]. This would explain why statins fail to consistently result in reduction of MAU in subjects with low grade MAU, or why higher vs. lower doses of statin fail to further reduce MAU [29], [30], as the beneficial effect of statins on the glomerular MAU is counterbalanced by the induction of tubular MAU. It is very unlikely that statin induced MAU is associated with an increased cardiovascular risk, but its impact on the functional capacity and the morphological integrity of the kidneys is unknown. Even when the tubular albuminuria induced by statins is harmless [31], it interferes with the implication of MAU as predictor or marker of cardiovascular and renal risk by incorrectly labeling a subject as having a risk factor. Of note, this would imply that the prognostic impact of glomerular MAU (so not induced by statin use) in populations with a high prevalence of statin use, would be underestimated. The hypothesis that statin induced MAU is tubular in origin would also fit with the favorable effect of statins on cardiovascular disease and on progression of renal disease, as these effects are related to the reduction of glomerular MAU associated with the improvement of endothelial dysfunction [32], [33].

### Limitations

This cross-sectional study could not prove a causal connection between statin use and de novo MAU. Using the technique of propensity score we achieved “pseudo-randomization”, which in fact obviates the drawbacks imposed by the method of the study, but which is not free from unmeasured biases [24]. Unfortunately, although dose dependency and a higher frequency of more potent inhibitors of HMG coA reductase could add further strength to the association with MAU, the prescribed daily dose and type of statin were as per protocol not registered in our study. Another limitation of our study is that MAU was measured in a single morning urine, whereas guidelines recommend to have at least two positive MAU in three consecutive first morning urine samples before labeling a person as having MAU [34]. However, in view of the mechanism of inhibition of tubular endocytosis, it is unlikely that statin associated MAU would disappear by repeated testing, unless the statin would be stopped temporarily to confirm the diagnosis. Second, intermittent MAU should not be considered as a predictor or marker of cardiovascular or renal risk, as it is not linked to endothelial dysfunction [35]. In line with this, we demonstrated in another cohort, that patients with intermittent MAU have far less cardiovascular risk factors as compared to patients with persisting MAU [36]. Our study shows that, in addition to the problems caused by single vs. multiple sampling, the use of a statin can also lead to an in incorrect labeling of subject as having MAU.

The strength of this study is that it reflects routine clinical practice in hypertensive patients. To the best of our knowledge, this is the first study pointing to an independent association between statin use and MAU, even after correction for bias by indication by the use of a propensity score, underlining the potential consequences of confounding induced by statin use on the interpretation of MAU as a predictor or marker of cardiovascular or renal disease in epidemiological trials.

According to our data, statins are independently associated with an increased prevalence of MAU, even after correction for bias by indication. As this MAU is most likely of tubular origin, it is uncertain and rather unlikely whether it has the same prognostic impact for renal and cardiovascular disease as endothelial dysfunction induced glomerular MAU. As such, it can lead to incorrect labeling of subjects as having a cardiovascular risk factor.

## Supporting Information

Figure S1 **Flow chart of hypotheses to explain the observation of higher prevalence of MAU in statin users.** MAU: microalbuminuria, OR: odds ratio, CI: confidence interval.
(TIF)

Appendix Table S1 **List of missing data per variable.** ARB: angiotensin receptor blocker; ACE-I: angiotensin converting enzyme inhibitor.
(DOC)

Appendix Table S2 **Comparison of Baseline Characteristics Adjusted for Propensity Scores.** MS: metabolic syndrome; CV event: cardiovascular event; ARB: angiotensin receptor blocker; ACE-I: angiotensin converting enzyme inhibitor.
(DOC)

Appendix Table S3 **Propensity Score Model.** MS: metabolic syndrome; CV event: cardiovascular event; ARB: angiotensin receptor blocker.
(DOC)
