# Supplementary figures and images for: Statin Use and the Presence of Microalbuminuria. Results from the ERICABEL Trial: A Non-Interventional Epidemiological Cohort Study
Source: PLoS One. 2012 Feb 16;7(2):e31639. doi: 10.1371/journal.pone.0031639 (PMC3281099; doi:10.1371/journal.pone.0031639)

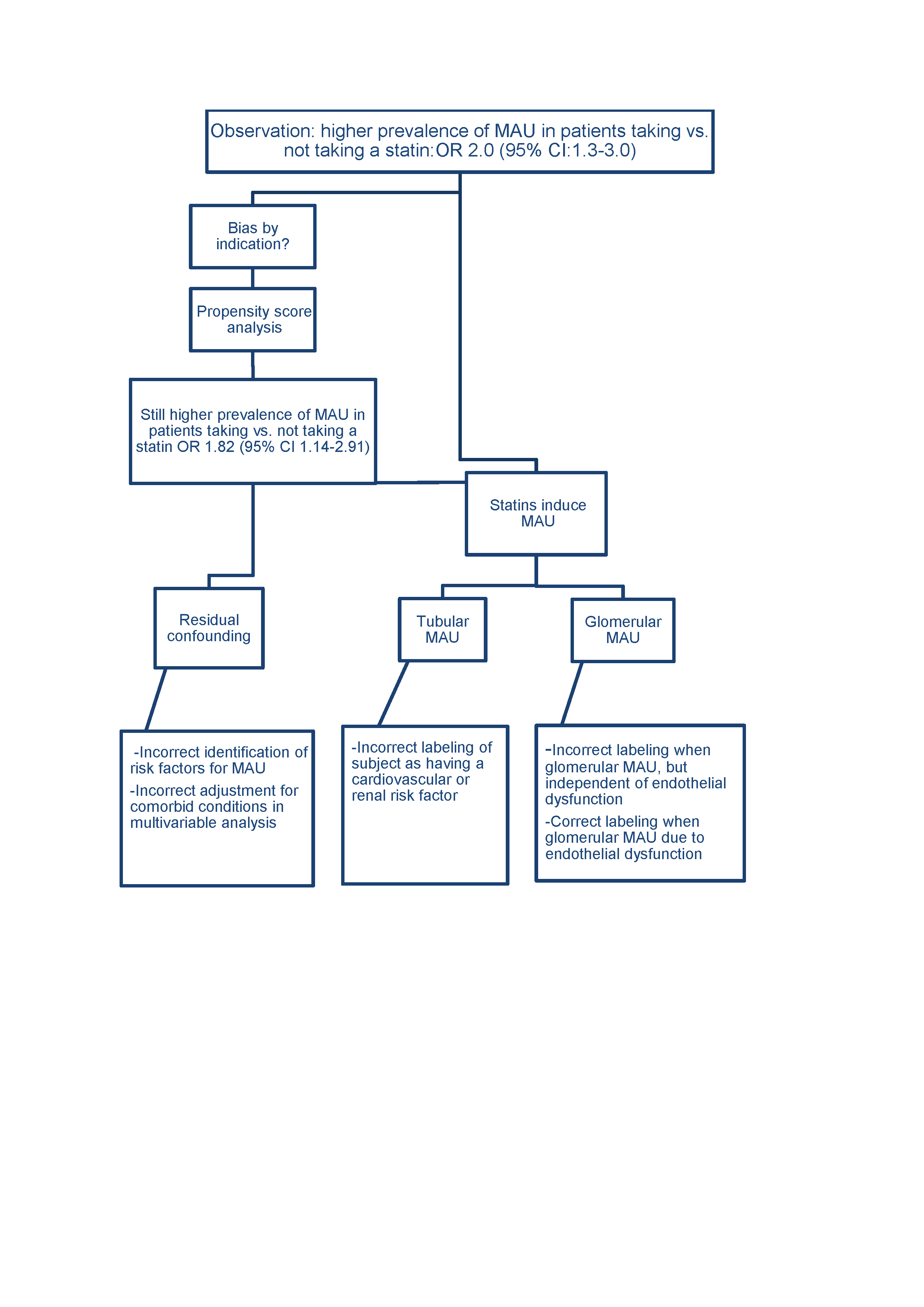

Supplement: Figure S1 — Flow chart of hypotheses to explain the observation of higher prevalence of MAU in statin users. MAU: microalbuminuria, OR: odds ratio, CI: confidence interval. (TIF) [file pone.0031639.s001.tif]
