# Supplementary material for: Statin Use and the Presence of Microalbuminuria. Results from the ERICABEL Trial: A Non-Interventional Epidemiological Cohort Study
Source: PLoS One. 2012 Feb 16;7(2):e31639. doi: 10.1371/journal.pone.0031639 (PMC3281099; doi:10.1371/journal.pone.0031639)
Supplement: Appendix Table S1 — List of missing data per variable. ARB: angiotensin receptor blocker; ACE-I: angiotensin converting enzyme inhibitor. (DOC) [file pone.0031639.s002.doc]

**Appendix table S1: list of missing data per variable**

| **Variable** | **Number of patients with missing** |
| --- | --- |
| Statin use | 20 |
| Microalbuminuria | 295 |
| Gender | 1 |
| Age | 1 |
| BMI | 56 |
| Systolic BP | 36 |
| Diastolic BP | 36 |
| Fasting glucose | 117 |
| Triglycerides | 118 |
| LDL cholesterol | 132 |
| C-Reactive protein | 173 |
| Serum uric acid | 137 |
| HDL cholesterol | 120 |
| ACE-I/ARB treatment | 20 |
| Cardiovascular event | 53 |
| Metabolic syndrome | 149 |
| Diabetes | 44 |
| Current smoking | 48 |
| **Any Missing** | **420** |
