# Supplementary material for: Statin Use and the Presence of Microalbuminuria. Results from the ERICABEL Trial: A Non-Interventional Epidemiological Cohort Study
Source: PLoS One. 2012 Feb 16;7(2):e31639. doi: 10.1371/journal.pone.0031639 (PMC3281099; doi:10.1371/journal.pone.0031639)
Supplement: Appendix Table S2 — Comparison of Baseline Characteristics Adjusted for Propensity Scores. MS: metabolic syndrome; CV event: cardiovascular event; ARB: angiotensin receptor blocker; ACE-I: angiotensin converting enzyme inhibitor. (DOC) [file pone.0031639.s003.doc]

| **Appendix Table S2: Comparison of Baseline Characteristics Adjusted for Propensity Scores** |
| --- |

| **1.1 Imputation 1** |
| --- |

|  | | | | | **P-value** | |
| --- | --- | --- | --- | --- | --- | --- |
| **Patient Characteristic** | **Statistic** | **No statins** | **Statins** | **Total** | **Unadjusted** | **Adjusted for Propensity Score** |
| **Male gender** | n/N (%) | 348/739 ( 47.09%) | 205/337 ( 60.83%) | 553/1076 ( 51.39%) | <.001 | 0.983 |
| **MS** | n/N (%) | 294/739 ( 39.78%) | 190/337 ( 56.38%) | 484/1076 ( 44.98%) | <.001 | 0.712 |
| **Diabetes** | n/N (%) | 101/739 ( 13.67%) | 106/337 ( 31.45%) | 207/1076 ( 19.24%) | <.001 | 0.383 |
| **Tobacco use** | n/N (%) | 261/739 ( 35.32%) | 130/337 ( 38.58%) | 391/1076 ( 36.34%) | 0.303 | 0.994 |
| **Microalbuminuria** | n/N (%) | 112/739 ( 15.16%) | 71/337 ( 21.07%) | 183/1076 ( 17.01%) | 0.017 | 0.230 |
| **ACE-i/ARB** | n/N (%) | 366/739 ( 49.53%) | 237/337 ( 70.33%) | 603/1076 ( 56.04%) | <.001 | 0.999 |
| **CV event** | n/N (%) | 43/739 ( 5.82%) | 81/337 ( 24.04%) | 124/1076 ( 11.52%) | <.001 | 0.783 |
|  | | | | | | |

| **Appendix Table S2: Comparison of Baseline Characteristics Adjusted for Propensity Scores** |
| --- |

| **1.1 Imputation 1** |
| --- |

|  | | | | | **P-value** | |
| --- | --- | --- | --- | --- | --- | --- |
| **Patient Characteristic** | **Statistic** | **No statins** | **Statins** | **Total** | **Unadjusted** | **Adjusted for Propensity Score** |
| **Age (years)** | Mean | 56.8±7.61 | 59.0±7.03 | 57.5±7.50 | <.001 | 0.975 |
|  | Median | 57.3 | 59.9 | 58.5 |  |  |
|  | (Q1, Q3) | (50.8; 63.0) | (54.1; 64.6) | (51.7; 63.3) |  |  |
| **Body Mass Index** | Mean | 29.35.34 | 30.6±5.55 | 29.7±5.44 | <.001 | 0.980 |
|  | Median | 28.5± | 29.7 | 28.8 |  |  |
|  | (Q1, Q3) | (25.5; 32.0) | (26.8; 33.7) | (25.9; 32.5) |  |  |
| **Mean systolic RR (mmHg)** | Mean | 143.5±15.39 | 142.8±16.45 | 143.3±15.72 | 0.480 | 0.996 |
|  | Median | 142.5 | 140.0 | 142.0 |  |  |
|  | (Q1, Q3) | (133.0; 152.5) | (130.0; 152.5) | (132.5; 152.5) |  |  |
| **Mean Diastolic RR (mmHg)** | Mean | 84.4±9.73 | 83.0±9.97 | 84.0±9.82 | 0.035 | 0.455 |
|  | Median | 84.0 | 82.5 | 83.5 |  |  |
|  | (Q1, Q3) | (78.5; 90.5) | (78.0; 89.5) | (78.0; 90.0) |  |  |
| **Fasting glucose** | Mean | 101.8±49.17 | 114.0±39.56 | 105.6±46.70 | <.001 | 0.978 |
|  | Median | 94.0 | 102.0 | 96.0 |  |  |
|  | (Q1, Q3) | (87.0; 105.0) | (91.0; 121.0) | (88.0; 109.0) |  |  |
| **Uric acid** | Mean | 5.7±1.53 | 6.0±1.43 | 5.8±1.51 | <.001 | 0.369 |
|  | Median | 5.6 | 6.0 | 5.7 |  |  |
|  | (Q1, Q3) | (4.6; 6.5) | (5.0; 7.0) | (4.7; 6.7) |  |  |

| **Appendix Table S2: Comparison of Baseline Characteristics Adjusted for Propensity Scores** |
| --- |

| **1.1 Imputation 1** |
| --- |

|  | | | | | **P-value** | |  |
| --- | --- | --- | --- | --- | --- | --- | --- |
| **Patient Characteristic** | **Statistic** | **No statins** | **Statins** | **Total** | **Unadjusted** | **Adjusted for Propensity Score** |  |
| **Triglycerides** | Mean | 146.3±98.85 | 167.1±94.99 | 152.9±98.09 | 0.001 | 0.983 |  |
|  | Median | 119.0 | 142.0 | 127.0 |  |  |  |
|  | (Q1, Q3) | (87.0; 175.0) | (101.0; 206.0) | (92.0; 189.0) |  |  |  |
| **LDL** | Mean | 124.2±33.12 | 104.1±36.32 | 117.935.39 | <.001 | 0.946 |  |
|  | Median | 123.0 | 102.0 | 117.0 |  |  |  |
|  | (Q1, Q3) | (104.0; 144.0) | (79.0; 126.0) | (96.0; 139.0) |  |  |  |
| **HDL** | Mean | 57.7±18.62 | 54.1±17.99 | 56.6±18.49 | 0.003 | 0.984 |  |
|  | Median | 55.0 | 52.0 | 53.0 |  |  |  |
|  | (Q1, Q3) | (45.0; 67.0) | (43.0; 63.0) | (45.0; 65.0) |  |  |  |
| **CRP** | Mean | 0.5±0.75 | 0.4±0.54 | 0.5±0.69 | 0.174 | 0.993 |  |
|  | Median | 0.3 | 0.2 | 0.3 |  |  |  |
|  | (Q1, Q3) | (0.1; 0.6) | (0.1; 0.5) | (0.1; 0.5) |  |  |  |
|  | | | | | | | |

| **Appendix Table S2: Comparison of Baseline Characteristics Adjusted for Propensity Scores** |
| --- |

| **1.2 Imputation 2** |
| --- |

|  | | | | | **P-value** | |
| --- | --- | --- | --- | --- | --- | --- |
| **Patient Characteristic** | **Statistic** | **No statins** | **Statins** | **Total** | **Unadjusted** | **Adjusted for Propensity Score** |
| Male gender | n/N (%) | 349/739 ( 47.23%) | 203/337 ( 60.24%) | 552/1076 ( 51.30%) | <.001 | 0.991 |
| **MS** | n/N (%) | 284/739 ( 38.43%) | 196/337 ( 58.16%) | 480/1076 ( 44.61%) | <.001 | 0.718 |
| **Type II Diabetes** | n/N (%) | 103/739 ( 13.94%) | 106/337 ( 31.45%) | 209/1076 ( 19.42%) | <.001 | 0.318 |
| **Tobacco use** | n/N (%) | 265/739 ( 35.86%) | 129/337 ( 38.28%) | 394/1076 ( 36.62%) | 0.445 | 0.998 |
| **Microalbuminuria** | n/N (%) | 98/739 ( 13.26%) | 86/337 ( 25.52%) | 184/1076 ( 17.10%) | <.001 | <.001 |
| **ACE-i/ARB** | n/N (%) | 368/739 ( 49.80%) | 235/337 ( 69.73%) | 603/1076 ( 56.04%) | <.001 | 0.998 |
| **CV event** | n/N (%) | 43/739 ( 5.82%) | 81/337 ( 24.04%) | 124/1076 ( 11.52%) | <.001 | 0.866 |
|  | | | | | | |

| **Appendix Table S2: Comparison of Baseline Characteristics Adjusted for Propensity Scores** |
| --- |

| **1.2 Imputation 2** |
| --- |

|  | | | | | **P-value** | |
| --- | --- | --- | --- | --- | --- | --- |
| **Patient Characteristic** | **Statistic** | **No statins** | **Statins** | **Total** | **Unadjusted** | **Adjusted for Propensity Score** |
| **Age (years)** | Mean | 56.8±7.61 | 59.0±7.05 | 57.5±7.50 | <.001 | 0.987 |
|  | Median | 57.3 | 59.9 | 58.5 |  |  |
|  | (Q1, Q3) | (50.9; 63.0) | (54.1; 64.6) | (51.7; 63.4) |  |  |
| **Body Mass Index** | Mean | 29.3±5.36 | 30.5±5.48 | 29.7±5.43 | <.001 | 0.990 |
|  | Median | 28.5 | 29.7 | 28.8 |  |  |
|  | (Q1, Q3) | (25.5; 32.0) | (26.9; 33.6) | (25.9; 32.5) |  |  |
| **Mean Systolic RR (mmHg)** | Mean | 143.6±15.70 | 142.5±16.30 | 143.3±15.89 | 0.282 | 0.997 |
|  | Median | 142.5 | 140.0 | 142.0 |  |  |
|  | (Q1, Q3) | (132.5; 152.5) | (130.0; 152.0) | (131.5; 152.5) |  |  |
| **Mean Diastolic RR (mmHg)** | Mean | 84.5±9.72 | 82.8±9.82 | 83.9±9.78 | 0.012 | 0.195 |
|  | Median | 84.5 | 82.0 | 83.5 |  |  |
|  | (Q1, Q3) | (79.0; 90.5) | (77.5; 89.0) | (78.0; 90.0) |  |  |
| **Fasting glucose** | Mean | 101.6±48.69 | 113.6±38.95 | 105.4±46.18 | <.001 | 0.989 |
|  | Median | 95.0 | 102.0 | 96.0 |  |  |
|  | (Q1, Q3) | (87.0; 105.0) | (91.0; 119.0) | (88.0; 109.0) |  |  |
| **Uric acid** | Mean | 5.7±1.52 | 6.0±1.42 | 5.8±1.49 | 0.003 | 0.750 |
|  | Median | 5.6 | 5.9 | 5.7 |  |  |
|  | (Q1, Q3) | (4.6; 6.5) | (5.0; 6.9) | (4.7; 6.7) |  |  |
|  | | | | | | |

| **Appendix Table S2: Comparison of Baseline Characteristics Adjusted for Propensity Scores** |
| --- |

| **1.2 Imputation 2** |
| --- |

|  | | | | | **P-value** | |
| --- | --- | --- | --- | --- | --- | --- |
| **Patient Characteristic** | **Statistic** | **No statins** | **Statins** | **Total** | **Unadjusted** | **Adjusted for Propensity Score** |
| **Triglycerides** | Mean | 146.8±98.54 | 169.1±96.97 | 153.8±98.55 | <.001 | 0.990 |
|  | Median | 120.0 | 146.0 | 128.5 |  |  |
|  | (Q1, Q3) | (87.0; 176.0) | (101.0; 208.0) | (92.0; 189.0) |  |  |
| **LDL** | Mean | 124.5±33.10 | 103.3±36.50 | 117.8±35.57 | <.001 | 0.970 |
|  | Median | 123.0 | 101.0 | 117.0 |  |  |
|  | (Q1, Q3) | (105.0; 144.0) | (79.0; 125.0) | (96.0; 139.0) |  |  |
| **HDL** | Mean | 57.5±18.60 | 54.0±18.18 | 56.4±18.53 | 0.004 | 0.992 |
|  | Median | 55.0 | 51.0 | 53.0 |  |  |
|  | (Q1, Q3) | (45.0; 67.0) | (42.0; 63.0) | (44.0; 65.0) |  |  |
| **CRP** | Mean | 0.5±0.81 | 0.4±0.51 | 0.5±0.73 | 0.022 | 0.994 |
|  | Median | 0.3 | 0.2 | 0.3 |  |  |
|  | (Q1, Q3) | (0.1; 0.6) | (0.1; 0.5) | (0.1; 0.6) |  |  |
|  | | | | | | |

| **Appendix Table S2: Comparison of Baseline Characteristics Adjusted for Propensity Scores** |
| --- |

| **1.3 Imputation 3** |
| --- |

|  | | | | | **P-value** | |
| --- | --- | --- | --- | --- | --- | --- |
| **Patient Characteristic** | **Statistic** | **No statins** | **Statins** | **Total** | **Unadjusted** | **Adjusted for Propensity Score** |
| **Male gender** | n/N (%) | 347/737 ( 47.08%) | 206/339 ( 60.77%) | 553/1076 ( 51.39%) | <.001 | 0.991 |
| **MS** | n/N (%) | 290/737 ( 39.35%) | 194/339 ( 57.23%) | 484/1076 ( 44.98%) | <.001 | 0.969 |
| **Type II Diabetes** | n/N (%) | 103/737 ( 13.98%) | 107/339 ( 31.56%) | 210/1076 ( 19.52%) | <.001 | 0.335 |
| **tobacco use** | n/N (%) | 261/737 ( 35.41%) | 130/339 ( 38.35%) | 391/1076 ( 36.34%) | 0.353 | 0.996 |
| **Microalbuminuria** | n/N (%) | 102/737 ( 13.84%) | 89/339 ( 26.25%) | 191/1076 ( 17.75%) | <.001 | <.001 |
| **ACE-i/ARB** | n/N (%) | 361/737 ( 48.98%) | 235/339 ( 69.32%) | 596/1076 ( 55.39%) | <.001 | 0.988 |
| **CV event** | n/N (%) | 40/737 ( 5.43%) | 82/339 ( 24.19%) | 122/1076 ( 11.34%) | <.001 | 0.751 |
|  | | | | | | |

| **Appendix Table S2: Comparison of Baseline Characteristics Adjusted for Propensity Scores** |
| --- |

| **1.3 Imputation 3** |
| --- |

|  | | | | | **P-value** | |
| --- | --- | --- | --- | --- | --- | --- |
| **Patient Characteristic** | **Statistic** | **No statins** | **Statins** | **Total** | **Unadjusted** | **Adjusted for Propensity Score** |
| **Age (years** | Mean | 56.8±7.61 | 59.0±7.04 | 57.5±7.51 | <.001 | 0.982 |
|  | Median | 57.3 | 59.9 | 58.5 |  |  |
|  | (Q1, Q3) | (50.8; 62.9) | (54.1; 64.6) | (51.7; 63.3) |  |  |
| **Body Mass Index** | Mean | 29.2±5.32 | 30.6±5.49 | 29.7±5.41 | <.001 | 0.985 |
|  | Median | 28.5 | 29.7 | 28.8 |  |  |
|  | (Q1, Q3) | (25.6; 32.0) | (26.9; 33.7) | (25.9; 32.5) |  |  |
| **Mean Systolic RR (mmHg)** | Mean | 143.3±15.34 | 142.6±16.26 | 143.1±15.63 | 0.483 | 0.997 |
|  | Median | 142.0 | 140.0 | 142.0 |  |  |
|  | (Q1, Q3) | (132.5; 152.5) | (130.0; 152.0) | (132.3; 152.5) |  |  |
| **Mean Diastolic RR (mmHg)** | Mean | 84.3±9.67 | 83.0±9.82 | 83.9±9.73 | 0.037 | 0.359 |
|  | Median | 84.5 | 82.0 | 83.5 |  |  |
|  | (Q1, Q3) | (78.5; 90.0) | (78.0; 89.5) | (78.5; 90.0) |  |  |
| **Fasting glucose** | Mean | 101.6±48.67 | 116.6±73.03 | 106.4±57.86 | <.001 | 0.985 |
|  | Median | 94.0 | 102.0 | 96.0 |  |  |
|  | (Q1, Q3) | (87.0; 105.0) | (91.0; 119.0) | (88.0; 109.0) |  |  |
| **Uric acid** | Mean | 5.7±1.51 | 6.0±1.40 | 5.8±1.49 | 0.003 | 0.735 |
|  | Median | 5.6 | 6.0 | 5.7 |  |  |
|  | (Q1, Q3) | (4.6; 6.5) | (4.9; 6.9) | (4.7; 6.7) |  |  |
|  | | | | | | |

| **Appendix Table S2: Comparison of Baseline Characteristics Adjusted for Propensity Scores** |
| --- |

| **1.3 Imputation 3** |
| --- |

|  | | | | | **P-value** | |
| --- | --- | --- | --- | --- | --- | --- |
| **Patient Characteristic** | **Statistic** | **No statins** | **Statins** | **Total** | **Unadjusted** | **Adjusted for Propensity Score** |
| **Triglycerides** | Mean | 146.2±97.55 | 169.4±96.29 | 153.6±97.71 | <.001 | 0.986 |
|  | Median | 120.0 | 147.0 | 128.0 |  |  |
|  | (Q1, Q3) | (87.0; 176.0) | (101.0; 208.0) | (92.0; 190.0) |  |  |
| **LDL** | Mean | 124.5±32.96 | 103.4±36.80 | 117.9±35.58 | <.001 | 0.959 |
|  | Median | 123.0 | 101.0 | 117.0 |  |  |
|  | (Q1, Q3) | (104.0; 144.0) | (78.0; 125.0) | (96.0; 139.0) |  |  |
| **HDL** | Mean | 57.9±18.56 | 53.9±17.67 | 56.6±18.37 | <.001 | 0.987 |
|  | Median | 55.0 | 51.0 | 53.0 |  |  |
|  | (Q1, Q3) | (46.0; 67.0) | (43.0; 63.0) | (45.0; 65.0) |  |  |
| **CRP** | Mean | 0.5±0.82 | 0.4±0.52 | 0.5±0.74 | 0.026 | 0.992 |
|  | Median | 0.3 | 0.2 | 0.3 |  |  |
|  | (Q1, Q3) | (0.1; 0.6) | (0.1; 0.5) | (0.1; 0. 6) |  |  |
|  | | | | | | |

|  |  |
| --- | --- |
| **Appendix Table S2: Comparison of Baseline Characteristics Adjusted for Propensity Scores** | |

| **1.4 Imputation 4** |
| --- |

|  | | | | | **P-value** | |
| --- | --- | --- | --- | --- | --- | --- |
| **Patient Characteristic** | **Statistic** | **No statins** | **Statins** | **Total** | **Unadjusted** | **Adjusted for Propensity Score** |
| **Male gender** | n/N (%) | 346/736 ( 47.01%) | 207/340 ( 60.88%) | 553/1076 ( 51.39%) | <.001 | 0.971 |
| **MS** | n/N (%) | 286/736 ( 38.86%) | 196/340 ( 57.65%) | 482/1076 ( 44.80%) | <.001 | 0.617 |
| **Type II Diabetes** | n/N (%) | 100/736 ( 13.59%) | 107/340 ( 31.47%) | 207/1076 ( 19.24%) | <.001 | 0.301 |
| **Tobacco use** | n/N (%) | 264/736 ( 35.87%) | 128/340 ( 37.65%) | 392/1076 ( 36.43%) | 0.573 | 0.996 |
| **Microalbuminuria** | n/N (%) | 101/736 ( 13.72%) | 72/340 ( 21.18%) | 173/1076 ( 16.08%) | 0.002 | 0.026 |
| **ACE-i/ARB** | n/N (%) | 364/736 ( 49.46%) | 236/340 ( 69.41%) | 600/1076 ( 55.76%) | <.001 | 0.969 |
| **CV event** | n/N (%) | 41/736 ( 5.57%) | 82/340 ( 24.12%) | 123/1076 ( 11.43%) | <.001 | 0.799 |
|  | | | | | | |

| **Appendix Table S2: Comparison of Baseline Characteristics Adjusted for Propensity Scores** |
| --- |

| **1.4 Imputation 4** |
| --- |

|  | | | | | **P-value** | |
| --- | --- | --- | --- | --- | --- | --- |
| **Patient Characteristic** | **Statistic** | **No statins** | **Statins** | **Total** | **Unadjusted** | **Adjusted for Propensity Score** |
| **Age (years)** | Mean | 56.8±7.62 | 59.0±7.01 | 57.5±7.50 | <.001 | 0.966 |
|  | Median | 57.2 | 59.9 | 58.5 |  |  |
|  | (Q1, Q3) | (50.8; 63.0) | (54.1; 64.6) | (51.7; 63.3) |  |  |
| **Body Mass Index** | Mean | 29.2±5.31 | 30.5±5.50 | 29.7±5.40 | <.001 | 0.973 |
|  | Median | 28.5 | 29.7 | 28.7 |  |  |
|  | (Q1, Q3) | (25.6; 32.0) | (26.8; 33.6) | (26.0; 32.5) |  |  |
| **Mean Systolic RR (mmHg)** | Mean | 143.4±15.44 | 142.7±16.24 | 143.2±15.69 | 0.477 | 0.995 |
|  | Median | 142.5 | 140.0 | 142.0 |  |  |
|  | (Q1, Q3) | (132.5; 152.5) | (130.0; 152.3) | (131.8; 152.5) |  |  |
| **Mean Diastolic RR (mmHg)** | Mean | 84.4±9.65 | 83.0±9.89 | 84.0±9.75 | 0.026 | 0.408 |
|  | Median | 84.5 | 82.3 | 83.5 |  |  |
|  | (Q1, Q3) | (79.0; 90.5) | (78.0; 89.5) | (78.5; 90.0) |  |  |
| **Fasting glucose** | Mean | 101.1±48.75 | 113.1±36.39 | 104.9±45.54 | <.001 | 0.970 |
|  | Median | 94.0 | 102.0 | 96.0 |  |  |
|  | (Q1, Q3) | (87.0; 104.0) | (91.0; 120.5) | (88.0; 109.0) |  |  |
| **Uric acid** | Mean | 5.7±1.52 | 6.0±1.42 | 5.8±1.49 | 0.003 | 0.570 |
|  | Median | 5.7 | 6.0 | 5.7 |  |  |
|  | (Q1, Q3) | (4.7; 6.7) | (5.0; 7.0) | (4.7; 6.7) |  |  |
|  | | | | | | |

| **Appendix Table S2: Comparison of Baseline Characteristics Adjusted for Propensity Scores** |
| --- |

| **1.4 Imputation 4** |
| --- |

|  | | | | | **P-value** | |
| --- | --- | --- | --- | --- | --- | --- |
| **Patient Characteristic** | **Statistic** | **No statins** | **Statins** | **Total** | **Unadjusted** | **Adjusted for Propensity Score** |
| **Triglycerides** | Mean | 145.7±97.04 | 165.9±94.44 | 152.1±96.64 | 0.001 | 0.977 |
|  | Median | 120.0 | 144.0 | 128.0 |  |  |
|  | (Q1, Q3) | (86.0; 176.0) | (100.0; 203.0) | (91.0; 189.0) |  |  |
| **LDL** | Mean | 124.9±33.18 | 103.7±36.41 | 118.2±35.61 | <.001 | 0.923 |
|  | Median | 123.6 | 101.0 | 117.0 |  |  |
|  | (Q1, Q3) | (105.0; 145.0) | (79.0; 125.0) | (95.5; 139.0) |  |  |
| **HDL** | Mean | 57.7±18.52 | 54.3±17.98 | 56.7±18.41 | 0.005 | 0.980 |
|  | Median | 55.0 | 51.5 | 53.0 |  |  |
|  | (Q1, Q3) | (45.5; 67.0) | (43.0; 62.5) | (45.0; 65.0) |  |  |
| **C RP** | Mean | 0.5±0.77 | 0.4±0.52 | 0.5±0.70 | 0.060 | 0.986 |
|  | Median | 0.3 | 0.2 | 0.3 |  |  |
|  | (Q1, Q3) | (0.1; 0.6) | (0.1; 0.5) | (0.1; 0.6) |  |  |
|  | | | | | | |

| **Appendix Table S2: Comparison of Baseline Characteristics Adjusted for Propensity Scores** |
| --- |

| **1.5 Imputation 5** |
| --- |

|  | | | | | **P-value** | |
| --- | --- | --- | --- | --- | --- | --- |
| **Patient Characteristic** | **Statistic** | **No statins** | **Statins** | **Total** | **Unadjusted** | **Adjusted for Propensity Score** |
| **Male gender** | n/N (%) | 350/740 ( 47.30%) | 203/336 ( 60.42%) | 553/1076 ( 51.39%) | <.001 | 0.992 |
| **MS** | n/N (%) | 298/740 ( 40.27%) | 193/336 ( 57.44%) | 491/1076 ( 45.63%) | <.001 | 0.953 |
| **Type II Diabetes** | n/N (%) | 100/740 ( 13.51%) | 106/336 ( 31.55%) | 206/1076 ( 19.14%) | <.001 | 0.124 |
| **Current smoking** | n/N (%) | 267/740 ( 36.08%) | 128/336 ( 38.10%) | 395/1076 ( 36.71%) | 0.525 | 0.997 |
| **Microalbuminuria** | n/N (%) | 96/740 ( 12.97%) | 85/336 ( 25.30%) | 181/1076 ( 16.82%) | <.001 | <.001 |
| **ACE-i/ARB** | n/N (%) | 366/740 ( 49.46%) | 235/336 ( 69.94%) | 601/1076 ( 55.86%) | <.001 | 0.979 |
| **CV event** | n/N (%) | 43/740 ( 5.81%) | 84/336 ( 25.00%) | 127/1076 ( 11.80%) | <.001 | 0.720 |
|  | | | | | | |

| **Appendix Table S2: Comparison of Baseline Characteristics Adjusted for Propensity Scores** |
| --- |

| **1.5 Imputation 5** |
| --- |

|  | | | | | **P-value** | |
| --- | --- | --- | --- | --- | --- | --- |
| **Patient Characteristic** | **Statistic** | **No statins** | **Statins** | **Total** | **Unadjusted** | **Adjusted for Propensity Score** |
| **Age (years)** | Mean | 56.8±7.61 | 59.1±7.03 | 57.5±7.50 | <.001 | 0.981 |
|  | Median | 57.3 | 60.0 | 58.5 |  |  |
|  | (Q1, Q3) | (50.8; 63.0) | (54.1; 64.6) | (51.7; 63.3) |  |  |
| **Body Mass Index** | Mean | 29.3±5.30 | 30.5±5.51 | 29.7±5.39 | <.001 | 0.986 |
|  | Median | 28.5 | 29.5 | 28.7 |  |  |
|  | (Q1, Q3) | (25.7; 32.0) | (26.7; 33.6) | (26.0; 32.5) |  |  |
| **Mean Systolic RR (mmHg)** | Mean | 143.4±15.41 | 142.7±16.27 | 143.2±15.68 | 0.475 | 0.997 |
|  | Median | 142.5 | 140.0 | 142.0 |  |  |
|  | (Q1, Q3) | (132.5; 152.5) | (130.0; 152.3) | (131.8; 152.5) |  |  |
| **Mean Diastolic RR (mmHg** | Mean | 84.4±9.65 | 82.9±9.81 | 83.9±9.72 | 0.021 | 0.293 |
|  | Median | 84.3 | 82.3 | 83.5 |  |  |
|  | (Q1, Q3) | (79.0; 90.5) | (78.0; 89.3) | (78.5; 90.0) |  |  |
| **Fasting glucose** | Mean | 101.6±48.95 | 113.0±37.43 | 105.2±45.96 | <.001 | 0.984 |
|  | Median | 94.0 | 100.0 | 96.0 |  |  |
|  | (Q1, Q3) | (87.0; 105.0) | (91.0; 119.0) | (88.0; 109.0) |  |  |
| **Uric acid** | Mean | 5.7±1.51 | 6.0±1.42 | 5.8±1.49 | 0.004 | 0.476 |
|  | Median | 5.6 | 6.0 | 5.7 |  |  |
|  | (Q1, Q3) | (4.6; 6.6) | (4.9; 7.0) | (4.7; 6.7) |  |  |
|  | | | | | | |

| **Appendix Table S2: Comparison of Baseline Characteristics Adjusted for Propensity Scores** |
| --- |

| **1.5 Imputation 5** |
| --- |

|  | | | | | **P-value** | |
| --- | --- | --- | --- | --- | --- | --- |
| **Patient Characteristic** | **Statistic** | **No statins** | **Statins** | **Total** | **Unadjusted** | **Adjusted for Propensity Score** |
| **Triglycerides** | Mean | 146.6±99.35 | 168.1±96.71 | 153.3±98.99 | <.001 | 0.986 |
|  | Median | 119.5 | 144.0 | 127.0 |  |  |
|  | (Q1, Q3) | (87.0; 175.0) | (100.5; 205.5) | (92.0; 188.5) |  |  |
| **LDL** | Mean | 124.6±33.36 | 103.1±36.58 | 117.9±35.79 | <.001 | 0.956 |
|  | Median | 123.6 | 100.5 | 117.0 |  |  |
|  | (Q1, Q3) | (105.0; 145.0) | (78.5; 125.0) | (95.0; 140.0) |  |  |
| **HDL** | Mean | 57.8±18.66 | 54.6±18.54 | 56.8±18.68 | 0.009 | 0.989 |
|  | Median | 55.0 | 52.0 | 53.0 |  |  |
|  | (Q1, Q3) | (46.0; 67.0) | (43.0; 63.0) | (45.0; 65.0) |  |  |
| **CRP** | Mean | 0.5±0.87 | 0.4±0.67 | 0.5±0.81 | 0.057 | 0.992 |
|  | Median | 0.3 | 0.2 | 0.3 |  |  |
|  | (Q1, Q3) | (0.1; 0.6) | (0.1; 0.5) | (0.1; 0.6) |  |  |
|  | | | | | | |
