# Supplementary material for: Statin Use and the Presence of Microalbuminuria. Results from the ERICABEL Trial: A Non-Interventional Epidemiological Cohort Study
Source: PLoS One. 2012 Feb 16;7(2):e31639. doi: 10.1371/journal.pone.0031639 (PMC3281099; doi:10.1371/journal.pone.0031639)
Supplement: Appendix Table S3 — Propensity Score Model. MS: metabolic syndrome; CV event: cardiovascular event; ARB: angiotensin receptor blocker. (DOC) [file pone.0031639.s004.doc]

| **Appendix Table S3: Propensity Score Model** |
| --- |

| **2.1 Imputation 1** |
| --- |

|  | **Odds Ratio** | |
| --- | --- | --- |
| **Parameter** | **Estimate** | **95% Confidence Interval** |
| Age (tertiles) | 0.922 | ( 0.845; 1.006) |
| age1 | 1.108 | ( 0.908; 1.351) |
| age2 | 0.709 | ( 0.301; 1.672) |
| gender Female vs Male | 1.232 | ( 0.856; 1.773) |
| Bmi (tertiles) | 1.027 | ( 0.862; 1.224) |
| bmi1 | 1.209 | ( 0.624; 2.345) |
| bmi2 | 0.453 | ( 0.070; 2.909) |
| Waist (tertiles) | 0.991 | ( 0.936; 1.049) |
| waist1 | 0.882 | ( 0.757; 1.028) |
| waist2 | 1.988 | ( 1.071; 3.689) |
| systolic blood pressure (tertiles) | 0.993 | ( 0.954; 1.033) |
| systolic blood pressure1 | 1.100 | ( 0.963; 1.257) |
| systolic blood pressure2 | 0.672 | ( 0.423; 1.069) |
| Smoke use No vs Yes | 0.850 | ( 0.610; 1.186) |
| Cv Event No vs Yes | 3.321 | ( 2.100; 5.251) |
| CRP | 172.681 | ( 5.599; >999.999) |
| Note: The propensity model models the probability of not receiving statins. All continuous variables in the model are included using restricted cubic splines. The c-statistic for the propensity model is 0.791. | | |

| **Appendix Table S3: Propensity Score Model** |
| --- |

| **2.1 Imputation 1** |
| --- |

|  | **Odds Ratio** | |
| --- | --- | --- |
| **Parameter** | **Estimate** | **95% Confidence Interval** |
| crp1 | <0.001 | ( <0.001; <0.001) |
| crp2 | >999.999 | (>999.999; >999.999) |
| Fasting glucose (tertiles) | 0.949 | ( 0.899; 1.002) |
| fasting glucose1 | 1.301 | ( 0.799; 2.118) |
| fasting glucose2 | 0.598 | ( 0.205; 1.744) |
| Hdl (tertiles) | 0.980 | ( 0.938; 1.023) |
| hdl1 | 0.997 | ( 0.822; 1.208) |
| hdl2 | 1.100 | ( 0.635; 1.907) |
| LDL (tertiles) | 1.022 | ( 1.007; 1.037) |
| ldl1 | 1.023 | ( 0.980; 1.067) |
| ldl2 | 0.838 | ( 0.702; 1.002) |
| Triglycerides (tertiles) | 0.989 | ( 0.976; 1.002) |
| triglycerides1 | 1.018 | ( 0.927; 1.117) |
| triglycerides2 | 0.990 | ( 0.816; 1.200) |
| ARB No vs Yes | 1.610 | ( 1.171; 2.213) |
| Note: The propensity model models the probability of not receiving statins. All continuous variables in the model are included using restricted cubic splines. The c-statistic for the propensity model is 0.791. | | |

| **Appendix Table S3: Propensity Score Model** |
| --- |

| **2.2 Imputation 2** |
| --- |

|  | **Odds Ratio** | |
| --- | --- | --- |
| **Parameter** | **Estimate** | **95% Confidence Interval** |
| Age | 0.936 | ( 0.858; 1.022) |
| age1 | 1.067 | ( 0.873; 1.303) |
| age2 | 0.823 | ( 0.347; 1.954) |
| gender Female vs Male | 1.158 | ( 0.801; 1.674) |
| Bmi | 1.035 | ( 0.871; 1.229) |
| bmi1 | 1.105 | ( 0.573; 2.129) |
| bmi2 | 0.589 | ( 0.093; 3.744) |
| Waist | 1.003 | ( 0.947; 1.061) |
| waist1 | 0.852 | ( 0.731; 0.993) |
| waist2 | 2.316 | ( 1.247; 4.300) |
| systolic blood pressure | 0.992 | ( 0.953; 1.032) |
| systolic blood pressure1 | 1.112 | ( 0.972; 1.272) |
| systolic blood pressure2 | 0.649 | ( 0.406; 1.037) |
| Smoke use No vs Yes | 0.843 | ( 0.604; 1.177) |
| Cv Event No vs Yes | 3.180 | ( 2.006; 5.042) |
| CRP | 182.914 | ( 5.879; >999.999) |
| Note: The propensity model models the probability of not receiving statins. All continuous variables in the model are included using restricted cubic splines. The c-statistic for the propensity model is 0.796. | | |

| **Appendix Table S3: Propensity Score Model** |
| --- |

| **2.3 Imputation 2** |
| --- |

|  | **Odds Ratio** | |
| --- | --- | --- |
| **Parameter** | **Estimate** | **95% Confidence Interval** |
| crp1 | <0.001 | ( <0.001; <0.001) |
| crp2 | >999.999 | (>999.999; >999.999) |
| fasting glucose | 0.946 | ( 0.895; 1.001) |
| fasting glucose1 | 1.369 | ( 0.830; 2.258) |
| fasting glucose2 | 0.531 | ( 0.177; 1.592) |
| HDL | 0.975 | ( 0.933; 1.020) |
| hdl1 | 1.038 | ( 0.854; 1.262) |
| hdl2 | 0.974 | ( 0.558; 1.697) |
| LDL | 1.022 | ( 1.007; 1.038) |
| ldl1 | 1.028 | ( 0.985; 1.073) |
| ldl2 | 0.814 | ( 0.680; 0.975) |
| Triglycerides | 0.990 | ( 0.977; 1.003) |
| triglycerides1 | 1.009 | ( 0.919; 1.108) |
| triglycerides2 | 1.007 | ( 0.830; 1.221) |
| ARB No vs Yes | 1.486 | ( 1.081; 2.044) |
| Note: The propensity model models the probability of not receiving statins. All continuous variables in the model are included using restricted cubic splines. The c-statistic for the propensity model is 0.796. | | |

| **Table S3: Propensity Score Model** |
| --- |

| **3.1 Imputation 3** |
| --- |

|  | **Odds Ratio** | |
| --- | --- | --- |
| **Parameter** | **Estimate** | **95% Confidence Interval** |
| Age | 0.944 | ( 0.865; 1.029) |
| age1 | 1.037 | ( 0.849; 1.265) |
| age2 | 0.940 | ( 0.398; 2.221) |
| gender Female vs Male | 1.226 | ( 0.849; 1.769) |
| Bmi | 1.066 | ( 0.900; 1.262) |
| bmi1 | 0.924 | ( 0.483; 1.769) |
| bmi2 | 1.006 | ( 0.161; 6.271) |
| Waist | 0.974 | ( 0.919; 1.033) |
| waist1 | 0.942 | ( 0.807; 1.099) |
| waist2 | 1.533 | ( 0.823; 2.855) |
| systolic blood pressure | 0.994 | ( 0.956; 1.034) |
| systolic blood pressure1 | 1.092 | ( 0.956; 1.248) |
| systolic blood pressure2 | 0.694 | ( 0.436; 1.103) |
| Smoke use No vs Yes | 0.837 | ( 0.600; 1.167) |
| Cv Event No vs Yes | 3.430 | ( 2.156; 5.457) |
| CRP | 258.881 | ( 8.160; >999.999) |
| Note: The propensity model models the probability of not receiving statins. All continuous variables in the model are included using restricted cubic splines. The c-statistic for the propensity model is 0.795. | | |

| **Appendix Table S3: Propensity Score Model** |
| --- |

| **3.2 Imputation 3** |
| --- |

|  | **Odds Ratio** | |
| --- | --- | --- |
| **Parameter** | **Estimate** | **95% Confidence Interval** |
| crp1 | <0.001 | ( <0.001; <0.001) |
| crp2 | >999.999 | (>999.999; >999.999) |
| Fasting glucuse | 0.950 | ( 0.900; 1.003) |
| fasting glucose1 | 1.339 | ( 0.822; 2.183) |
| fasting glucose2 | 0.556 | ( 0.190; 1.624) |
| HDL | 0.966 | ( 0.924; 1.009) |
| hdl1 | 1.087 | ( 0.895; 1.320) |
| hdl2 | 0.861 | ( 0.494; 1.500) |
| LDL | 1.025 | ( 1.009; 1.040) |
| ldl1 | 1.020 | ( 0.977; 1.065) |
| ldl2 | 0.839 | ( 0.700; 1.006) |
| Triglycerides | 0.991 | ( 0.978; 1.004) |
| triglycerides1 | 1.001 | ( 0.910; 1.101) |
| triglycerides2 | 1.025 | ( 0.842; 1.247) |
| ARB No vs Yes | 1.531 | ( 1.114; 2.104) |
| Note: The propensity model models the probability of not receiving statins. All continuous variables in the model are included using restricted cubic splines. The c-statistic for the propensity model is 0.795. | | |

| **Appendix Table S3: Propensity Score Model** |
| --- |

| **4.1 Imputation 4** |
| --- |

|  | **Odds Ratio** | |
| --- | --- | --- |
| **Parameter** | **Estimate** | **95% Confidence Interval** |
| Age | 0.945 | ( 0.866; 1.031) |
| age1 | 1.044 | ( 0.855; 1.274) |
| age2 | 0.908 | ( 0.384; 2.147) |
| gender Female vs Male | 1.181 | ( 0.817; 1.706) |
| Bmi | 1.050 | ( 0.883; 1.249) |
| bmi1 | 1.030 | ( 0.532; 1.995) |
| bmi2 | 0.737 | ( 0.115; 4.745) |
| Waist | 0.981 | ( 0.924; 1.041) |
| waist1 | 0.919 | ( 0.785; 1.077) |
| waist2 | 1.683 | ( 0.892; 3.176) |
| systolic blood pressure | 0.989 | ( 0.950; 1.030) |
| systolic blood pressure1 | 1.104 | ( 0.964; 1.264) |
| systolic blood pressure2 | 0.676 | ( 0.422; 1.084) |
| Smoke use No vs Yes | 0.816 | ( 0.583; 1.140) |
| Cv Event No vs Yes | 3.609 | ( 2.267; 5.743) |
| CRP | 161.595 | ( 5.242; >999.999) |
| Note: The propensity model models the probability of not receiving statins. All continuous variables in the model are included using restricted cubic splines. The c-statistic for the propensity model is 0.796. | | |

| **Appendix Table S3: Propensity Score Model** |
| --- |

| **4.2 Imputation 4** |
| --- |

|  | **Odds Ratio** | |
| --- | --- | --- |
| **Parameter** | **Estimate** | **95% Confidence Interval** |
| crp1 | <0.001 | ( <0.001; <0.001) |
| crp2 | >999.999 | (>999.999; >999.999) |
| Fasting glucose | 0.932 | ( 0.881; 0.987) |
| fasting glucose1 | 1.472 | ( 0.888; 2.441) |
| fasting glucose2 | 0.463 | ( 0.153; 1.403) |
| HDL | 0.972 | ( 0.928; 1.017) |
| hdl1 | 1.046 | ( 0.858; 1.276) |
| hdl2 | 0.962 | ( 0.547; 1.692) |
| LDL | 1.025 | ( 1.009; 1.041) |
| ldl1 | 1.017 | ( 0.973; 1.062) |
| ldl2 | 0.856 | ( 0.714; 1.026) |
| Triglycerides | 0.991 | ( 0.978; 1.004) |
| triglycerides1 | 1.013 | ( 0.923; 1.113) |
| triglycerides2 | 0.995 | ( 0.821; 1.206) |
| ARB No vs Yes | 1.575 | ( 1.147; 2.161) |
| Note: The propensity model models the probability of not receiving statins. All continuous variables in the model are included using restricted cubic splines. The c-statistic for the propensity model is 0.796. | | |

| **Appendix Table S3: Propensity Score Model** |
| --- |

| **5.1 Imputation 5** |
| --- |

|  | **Odds Ratio** | |
| --- | --- | --- |
| **Parameter** | **Estimate** | **95% Confidence Interval** |
| Age | 0.947 | ( 0.867; 1.034) |
| age1 | 1.045 | ( 0.855; 1.276) |
| age2 | 0.882 | ( 0.372; 2.088) |
| gender Female vs Male | 1.113 | ( 0.767; 1.615) |
| Bmi | 1.033 | ( 0.874; 1.221) |
| bmi1 | 1.140 | ( 0.598; 2.174) |
| bmi2 | 0.540 | ( 0.087; 3.342) |
| Waist | 0.988 | ( 0.933; 1.047) |
| waist1 | 0.902 | ( 0.773; 1.053) |
| waist2 | 1.795 | ( 0.963; 3.348) |
| systolic blood pressure | 0.990 | ( 0.952; 1.030) |
| systolic blood pressure1 | 1.104 | ( 0.966; 1.262) |
| systolic blood pressure2 | 0.672 | ( 0.422; 1.070) |
| Smoke use No vs Yes | 0.841 | ( 0.602; 1.173) |
| Cv Event No vs Yes | 3.396 | ( 2.145; 5.378) |
| CRP | 101.173 | ( 3.419; >999.999) |
| Note: The propensity model models the probability of not receiving statins. All continuous variables in the model are included using restricted cubic splines. The c-statistic for the propensity model is 0.791. | | |

| **Appendix Table S3: Propensity Score Model** |
| --- |

| **5.2 Imputation 5** |
| --- |

|  | **Odds Ratio** | |
| --- | --- | --- |
| **Parameter** | **Estimate** | **95% Confidence Interval** |
| crp1 | <0.001 | ( <0.001; <0.001) |
| crp2 | >999.999 | (>999.999; >999.999) |
| Fasting glucose | 0.933 | ( 0.883; 0.987) |
| fasting glucose1 | 1.534 | ( 0.933; 2.523) |
| fasting glucose2 | 0.416 | ( 0.140; 1.239) |
| HDL | 0.968 | ( 0.925; 1.013) |
| hdl1 | 1.078 | ( 0.884; 1.315) |
| hdl2 | 0.873 | ( 0.497; 1.533) |
| LDL | 1.020 | ( 1.005; 1.034) |
| ldl1 | 1.031 | ( 0.989; 1.075) |
| ldl2 | 0.811 | ( 0.679; 0.969) |
| Triglycerides | 0.991 | ( 0.978; 1.004) |
| triglycerides1 | 1.008 | ( 0.917; 1.107) |
| triglycerides2 | 1.008 | ( 0.830; 1.224) |
| ARB No vs Yes | 1.604 | ( 1.166; 2.207) |
| Note: The propensity model models the probability of not receiving statins. All continuous variables in the model are included using restricted cubic splines. The c-statistic for the propensity model is 0.791. | | |
